# Supplementary material for: A Multi-Disciplinary Team Approach to Genomic Testing for Drug-Resistant Epilepsy Patients—The GENIE Study
Source: J Clin Med. 2022 Jul 21;11(14):4238. doi: 10.3390/jcm11144238 (PMC9319736; doi:10.3390/jcm11144238)
Supplement: Supplementary file 1 [file jcm-11-04238-s001.zip › jcm-1808499-supplementary.pdf]

Integrating Epilepsy Genomics into Clinical Care- Patient Checklist

**Study ID**_____________<br>**Doctor's Name**_____________________________<br>**Hospital** ☐ RBWH ☐ QCH ☐ Cairns Base Hospital<br>**Date** _ _ / _ _ / _ _ _ _	Patient sticker

Eligibility checklist

Patient agrees to be contacted ☐ Yes ☐ No	Refractory epilepsy ☐ Yes ☐ No
No cause identified for epilepsy ☐ Yes ☐ No	Not pregnant ☐ Yes ☐ No

Patient **does not** have an idiopathic generalised epilepsy (highlighted over the page) ☐ Yes ☐ No
Patients does not require rapid genomic testing for acute clinical care decisions ☐ Yes ☐ No

If yes to all of the above, the patient is eligible and can be referred to this study. Please complete the clinical details.

Laboratory

**Extended Karyotype performed** ☐ Yes ☐ No<br>If yes- date performed _ _ / _ _ / _ _ _ _<br>If yes, results ☐ Normal ☐ Abnormal<br>If no- request form given ☐ Yes	**CMA performed** ☐ Yes ☐ No<br>If yes- date performed _ _ / _ _ / _ _ _ _<br>If yes, results ☐ Normal ☐ Abnormal<br>If no- request form given ☐ Yes

Other genetic testing performed ☐ Yes ☐ No If yes, results: ☐ Normal ☐ Abnormal
If yes, details: ____

Epilepsy/seizure details

**Age of onset:** ____________ (years)	**Handedness** ☐ Left ☐ Right ☐ Both ☐ Unknown

Focal seizure onset ☐ Yes ☐ No
If yes ☐ Aware ☐ Impaired awareness ☐ Motor onset ☐ Non-motor onset ☐ Focal to bilateral tonic-clonic

Generalised seizure onset ☐ Yes ☐ No
If yes ☐ Motor onset, Tonic-clonic ☐ Motor onset, other ☐ Non-motor onset (absence)

Unknown seizure onset ☐ Yes ☐ No
If yes ☐ Motor onset ☐ Non-motor onset ☐ Unclassified

Electroclinical syndrome/s, if known (list on pg. 2): ________________________________

Other clinical features – please tick all that apply

Dysmorphic features ☐ Yes ☐ No.
If yes, severity ☐ Mild ☐ Moderate ☐ Severe

Cognitive/Neurobehavioural ☐ Cognitive impairment ☐ Developmental delay ☐ Speech impairment
☐ Autistic spectrum disorder ☐ Attention deficit hyperactivity disorder ☐ Behavioural issues ☐ Other
If yes, severity ☐ Mild ☐ Moderate ☐ Severe If yes, age of onset: ____________ (years)

Regression ☐ Yes ☐ No ☐ Uncertain. If yes ☐ Cognitive ☐ Global
If yes, severity ☐ Mild ☐ Moderate ☐ Severe If yes, age of onset: ____________ (years)

Motor ☐ Cerebral Palsy
☐ Movement disorder, If yes ☐ Ataxia ☐ Other, specify: ______________________________
☐ Neuromuscular, If yes, specify: ____
If yes, severity ☐ Mild ☐ Moderate ☐ Severe

Other system involvement ☐ Eyes/vision ☐ Hearing ☐ Cardiac ☐ Other, specify: ____________________
____
If yes, severity ☐ Mild ☐ Moderate ☐ Severe

If yes to any of the clinical features, describe: ____
____

Integrating Epilepsy Genomics into Clinical Care- Patient Checklist

____

Investigations

**EEG** ☐ Yes ☐ No<br>Results ☐ Normal ☐ Abnormal<br>Describe findings: ________________________________<br>____	**Brain MRI** ☐ Yes ☐ No<br>Results ☐ Normal ☐ Abnormal<br>Describe findings: ________________________________<br>____

Family history

Family history of epilepsy ☐ Yes ☐ No

If yes, details (include relationship to patient and diagnosis): ______________________________________

____

____

Electroclinical Syndromes (adapted from Berg A.T. et al, 2010 Epilepsia)

(Highlighted phenotypes lack well-established genetic variants and will be generally excluded, but can be further discussed with the MDT)

Neonatal Period

- Benign familial neonatal epilepsy
- Early myoclonic encephalopathy
- Ohtahara syndrome

Infancy

- Epilepsy of infancy with migrating focal seizures
- West syndrome
- Myoclonic epilepsy in infancy
- Benign infantile epilepsy
- Benign familial infantile epilepsy
- Dravet syndrome
- Myoclonic encephalopathy in non-progressive disorders

Childhood

- Febrile seizures plus (can start in infancy)
- Panayiotopoulos syndrome
- Epilepsy with myoclonic atonic (previously "astatic") seizures
- Self-limiting epilepsy with centrotemporal spikes
- Autosomal dominant nocturnal frontal lobe epilepsy
- Late-onset childhood occipital epilepsy (Gastaut type)
- Epilepsy with myoclonic absences
- Lennox-Gastaut syndrome
- Epileptic encephalopathy with continuous spike-and-wave during sleep
- Landau-Kleffner syndrome
- Childhood absence epilepsy

Adolescence to Adulthood

- Juvenile absence epilepsy
- Juvenile myoclonic epilepsy
- Generalised tonic-clonic seizures alone
- Progressive myoclonic epilepsies
- Autosomal dominant epilepsy with auditory features
- Other familial temporal lobe epilepsies

Less Specific Age Relationships

- Familial focal epilepsy with variable foci (childhood to adult)
- Reflex epilepsies

Queensland Genomics   Queensland Government

ID: GENIE __ __ __

GENIE Project – Neurologist Survey (Pre-test)

Thank you for agreeing to participate in our study on the use of genomic testing in epilepsy care. We would appreciate it if you would complete this short questionnaire about your experience of genomic testing and about how you expect to use genomic testing in your clinical practice. This survey should take 2-5 minutes to complete.

Your participation is anonymous. The ethical aspects of this research project have been approved by Metro North Health Service District Human Research Ethics Committee, contact Manager, Research Ethics on 07 3646 5490. The Research Team, Carmen Bennett and Melanie Tom, (ph 07 3346 5027 or email: EpilepsyGenomics@health.qld.gov.au) are available to answer any questions you have about the study.

This survey is to be completed <u>before genomic testing results</u> have been returned to you for patients recruited into the GENIE Project.

Neurologist name: ______________________________   Date of survey completion: __ __/ __ __/ __ __ __ __

Clinical information

Patient: Date of birth: __ __/__ __/__ __ __ __   Sex: ☐ Female ☐ Male   UR: _______________

Facility: ☐ RBWH ☐ CHQ ☐ Cairns Hospital ☐ Other: _____________________________

1. How long has this patient been known to you? _________months ________years
2. What was the patient's age at seizure onset? _________years old ☐ I don't know

Pre-genomic management

3. What is the <u>most difficult</u> issue for this patient?
   (tick <u>one box only</u>)

   ☐ Seizure control
   ☐ Mood
   ☐ Not being able to drive
   ☐ Lack of diagnosis
   ☐ Not been able to be employed
   ☐ Poor memory
   ☐ Other: _______________________________

4. What are other difficult issues for this patient?
   (tick all that apply)

   ☐ Seizure control
   ☐ Poor mood
   ☐ Not being able to drive
   ☐ Lack of diagnosis
   ☐ Not been able to be employed
   ☐ Poor memory
   ☐ Other: _______________________________

5. Are you happy with the current drug treatment and seizure control?

   ☐ No ☐ Yes

6. What do you find is the hardest issue with the medication regime in this patient?

   ☐ Side-effects
   ☐ Lack of seizure control
   ☐ Lack of science with drug choice
   ☐ Compliance
   ☐ Other: _______________________________

7. What further investigations would you like to obtain for this patient to assist diagnosis?

   ☐ MR brain
   ☐ PET scan
   ☐ Video-EEG monitoring admission
   ☐ SPECT scan
   ☐ Other: ______________________
   ☐ None that I haven't already obtained

8. How often do you see this patient in the outpatient department?

- ☐ Monthly
- ☐ 3-monthly
- ☐ 6-monthy
- ☐ Yearly
- ☐ Other: ____________

9. What are the current obstacles to genomic testing for you? (tick all that apply)

- ☐ Cost (to patient or community)
- ☐ When to order
- ☐ How to order
- ☐ What to order
- ☐ Where to send the tests
- ☐ How to interpret reports
- ☐ What to say to patients
- ☐ Consenting the patients

Genomic integration into clinical care

10. Please rate how comfortable you are in integrating genomics into your clinical practice? (circle one number)

Least comfortable 1 2 3 4 5 6 7 Most comfortable

11. Please indicate how confident you are in your ability to do the following things.

	Very confident	Moderately confident	A little confident	Not confident at all
Ability to interpret genomic results in your disease area.	☐	☐	☐	☐
Ability to explain genomic concepts to patients.	☐	☐	☐	☐
Ability to make treatment recommendations based on genomic information.	☐	☐	☐	☐
Ability to provide genetic counselling support to the patient regarding their genomic results.	☐	☐	☐	☐
Ability to arrange most appropriate/effective genetic or genomic testing.	☐	☐	☐	☐

Thank you very much for completing this survey! Your participation is greatly appreciated.

ID: GENIE __ __ __

GENIE Project – Neurologist Survey (Post-test)

Thank you for agreeing to participate in our study on the use of genomic testing in epilepsy care. We would appreciate it if you would complete this short questionnaire about your experience of genomic testing and about how you expect to use genomic testing in your clinical practice. This survey should take 2-5 minutes to complete.

Your participation is anonymous. The ethical aspects of this research project have been approved by Metro North Health Service District Human Research Ethics Committee, contact Manager, Research Ethics on 07 3646 5490. The Research Team, Carmen Bennett and Melanie Tom, (ph 07 3346 5027 or email: EpilepsyGenomics@health.qld.gov.au) are available to answer any questions you have about the study.

This survey is to be completed 3 months after genomic testing results have been returned to you for patients recruited into the GENIE Project.

Neurologist name: ______________________________ Date of survey completion: __ __/ __ __/ __ __ __ __

Clinical information

Patient: Date of birth: __ __/__ __/__ __ __ __ Sex: ☐ Female ☐ Male UR: _______________

Facility: ☐ RBWH ☐ CHQ ☐ Cairns Hospital ☐ Other: _____________________________

Have genomic variants been identified? ☐ No ☐ Yes, please provide details here:

Post-genomic management

1. Has the genomic diagnosis impacted on your management?
   - ☐ Yes, definitely
   - ☐ Yes, somewhat
   - ☐ No, I did not find it clinically useful

2. Have the anti-epileptic medications changed?
   ☐ yes ☐ no
   If yes, please provide details here:

3. Have other management decisions changed (such as consideration for surgery, VNS or DBS) since you have received the results?
   ☐ yes ☐ no
   If yes, please provide details here:

4. Will you perform less investigations on this patient in the future?
   ☐ yes ☐ no
   If so, what will you do differently?

5. How often will you continue to see this patient in the outpatient department?
   - ☐ Monthly
   - ☐ 3-monthly
   - ☐ 6-monthy
   - ☐ Yearly
   - ☐ Other: ____________

6. Did you find the genomic report helpful?
   ☐ No ☐ Yes
   Please explain your response:

7. Did you attend a multi-disciplinary meeting?
   ☐ No ☐ Yes If so, was it helpful to you?
   ☐ No ☐ Yes
   Please explain your response:

8. Would you be interested in attending MDTs in the future?
   ☐ No ☐ Yes

9. Did you find the integration of a genetic counsellor helpful?
   ☐ No If no, why? __________________________
   ☐ Yes, if so, in what ways?
   - ☐ Helped provide options for patient management
   - ☐ Helped to consent patients
   - ☐ Helped with patient support
   - ☐ Helped with patient education
   - ☐ Helped with my education
   - ☐ Helped with discussion of results
   - ☐ Other: ___________________________________

10. Is there any other education/support/resources that would help you improve the integration of genomics into your practice?

Genomic integration into clinical care

11. Please rate how comfortable you are in integrating genomics into your clinical practice? (circle one number)

Least comfortable 1 2 3 4 5 6 7 Most comfortable

12. Please indicate how confident you are in your ability to do the following things.

	Very confident	Moderately confident	A little confident	Not confident at all
Ability to interpret genomic results in your disease area.	☐	☐	☐	☐
Ability to explain genomic concepts to patients.	☐	☐	☐	☐
Ability to make treatment recommendations based on genomic information.	☐	☐	☐	☐
Ability to provide genetic counselling support to the patient regarding their genomic results	☐	☐	☐	☐
Ability to arrange most appropriate/effective genetic or genomic testing.	☐	☐	☐	☐

Thank you very much for completing this survey! Your participation is greatly appreciated.
